# 1-Diazo­naphthalen-2(1*H*)-one

**DOI:** 10.1107/S1600536811026377

**Published:** 2011-07-09

**Authors:** Mitsuru Kitamura, Rie Sakata, Taisuke Matsumoto

**Affiliations:** a Department of Applied Chemistry, Kyushu Institute of Technology, 1-1 Sensuicho, Tobata, Kitakyushu 804-8550, Japan; b Institute for Materials Chemistry and Engineering, Kyushu University, 6-1, Kasugako-en, Kasuga 816-8580, Japan

## Abstract

The mol­ecule of the title compound, C_10_H_6_N_2_O, is nearly planar [maximum deviation = 0.030 (1) Å]. The CN_2_ moiety is almost linear, with a C—N—N angle of 175.50 (14)°. A single inter­molecular C—H⋯O hydrogen bond is observed in the crystal structure. A π–π inter­action is also observed with the shortest distance being 3.6832 (12) Å between the the centroids of the six-membered rings.

## Related literature

For the synthesis, see: Kitamura *et al.* (2010 ▶). For the crystal structure of related diazo­naphtho­quinones, see: Seidel *et al.* (1989 ▶); Ferreira *et al.* (2006 ▶). For an example of the utility of the diazo­naphtho­quinones, see Reiser *et al.* (1996 ▶). 
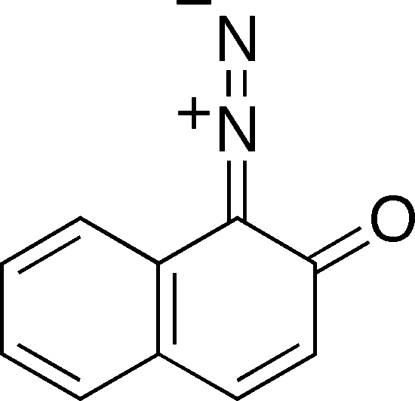


## Experimental

### 

#### Crystal data

C_10_H_6_N_2_O
*M* *_r_* = 170.17
Orthorhombic, 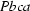

*a* = 11.900 (2) Å
*b* = 9.1978 (15) Å
*c* = 14.521 (3) Å
*V* = 1589.4 (5) Å^3^
*Z* = 8
Cu *K*α radiation
μ = 0.78 mm^−1^
*T* = 123 K
0.50 × 0.40 × 0.40 mm

#### Data collection

Rigaku R-AXIS RAPID diffractometer
Absorption correction: multi-scan (*ABSCOR*; Higashi, 1995 ▶) *T*_min_ = 0.566, *T*_max_ = 0.731
18038 measured reflections
1456 independent reflections
1359 reflections with *F* ^2^ > 2σ(*F* ^2^)
*R*_int_ = 0.018

#### Refinement

*R*[*F* ^2^ > 2σ(*F* ^2^)] = 0.044
*wR*(*F* ^2^) = 0.119
*S* = 1.08
1456 reflections
119 parameters
All H-atom parameters refined
Δρ_max_ = 0.29 e Å^−3^
Δρ_min_ = −0.13 e Å^−3^

### 

Data collection: *PROCESS-AUTO* (Rigaku, 1998 ▶); cell refinement: *PROCESS-AUTO*; data reduction: *CrystalStructure* (Rigaku Americas and Rigaku, 2007 ▶); program(s) used to solve structure: *SIR2002* (Burla *et al.*, 2003 ▶); program(s) used to refine structure: *SHELXL97* (Sheldrick, 2008 ▶); molecular graphics: *ORTEP-3* (Farrugia, 1997 ▶) and *Mercury* (Macrae *et al.*, 2008 ▶); software used to prepare material for publication: *CrystalStructure* and *publCIF* (Westrip, 2010 ▶).

## Supplementary Material

Crystal structure: contains datablock(s) global, I. DOI: 10.1107/S1600536811026377/pv2420sup1.cif

Structure factors: contains datablock(s) I. DOI: 10.1107/S1600536811026377/pv2420Isup2.hkl

Supplementary material file. DOI: 10.1107/S1600536811026377/pv2420Isup3.cml

Additional supplementary materials:  crystallographic information; 3D view; checkCIF report

## Figures and Tables

**Table 1:** Hydrogen-bond geometry (Å, °)

*D*—H⋯*A*	*D*—H	H⋯*A*	*D*⋯*A*	*D*—H⋯*A*
C6—H3⋯O1^i^	0.95	2.55	3.466 (2)	162

Symmetry code: (i) 
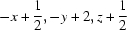
.

**Fig. 1.**
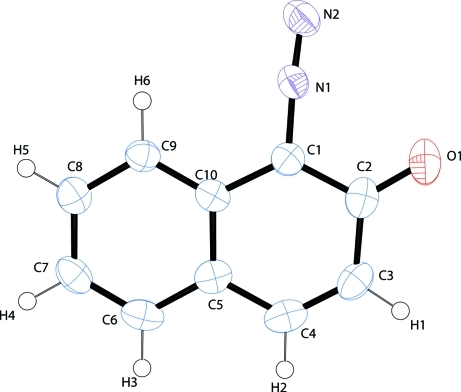
The molecular structure of the title compound with displacement ellipsoids drawn at the 50% probability level.

**Fig. 2.**
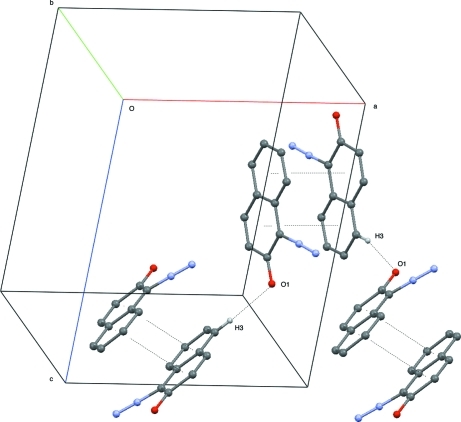
A unit cell packing diagram showing hydrogen bonds and π–π interaction; H-atoms not involved in H-bonds have been excluded for clarity.

## supplementary crystallographic information

### Comment

1,2-Diazonaphthoquinone derivatives are unique cyclic α-diazocarobonyl compounds that can be drawn as diazonium naphtholate resonance forms, and are exclusively used photoresists such as novolak-diazonaphthoquinone resist (Reiser, *et al.*, 1996). The reports on the X-ray structural data of diazonaphthoquinones are limitted (Seidel *et al.*, 1989; Ferreira *et al.*, 2006). We have synthesized the simplest diazonaphthoquinone, 1-diazo-1*H*-naphthalen-2-one, by the diazo-transfer reaction (Kitamura *et al.*, 2010) and determined its crystal structure which is being reported in this article.

In the structure of the title compound (Fig. 1) the CN_2_ moiety is almost linear, with C1—N1—N2 = 175.50 (14)°. The bond length N1—N2 and C1—N2 are 1.1210 (19) and 1.3355 (19) Å. The keto C═O bond length is 1.2474 (19) Å, which is close to a double bond. These data suggest that the structure of the title compound is not diazonium naphtholate form in the solid state.

A single intermolecular hydrogen bond is observed C6—H3···O1^i^ is observed in the crystal structure (Fig. 2).   In addition, a π –π interaction is obserbed with the shotest distance 3.6832 (12) Å between the the centroids of the six memberd rings.

### Experimental

To a solution of 2-chloro-1,3-dimethylimidazolinium chloride (228 mg, 1.35 mmol) in acetonitrile (2 ml), sodium azide (99.4 mg, 1.5 mmol) and 15-crown-5 ether (0.06 ml, 0.3 mmol) were added at 253 K and the mixture was stirred for 30 min. 2-Naphthol (130 mg, 0.90 mmol) and triethylamine (0.25 ml, 1.8 mmol) in THF (4 ml) were added to the mixture, which was stirred for 20 min. The reaction was quenched with water, and organic materials were extracted three times with CH_2_Cl_2_. The combined extracts were washed with water and brine, and then, dried over anhydrous sodium sulfate. The solvent was removed *in vacuo* to afford crude compound. The crude material was purified by flash column chromatography (silica gel: hexane/ethyl acetate = 4/1) to give the title compound in 86% yield. Single-crystals suitable for X-ray crystallographic analysis were obtained by recrystallization from a mixture of hexane and ethyl acetate (5:1).

### Refinement

H atoms were positioned geometrically and were refined in as riding mode on the parent atoms, with C–H distances of 0.95 Å and *U*_iso_(H) = 1.2*U*_eq_(C).

### Crystal data

**Table:**

C_10_H_6_N_2_O	*F*(000) = 704.00
*M**_r_* = 170.17	*D*_x_ = 1.422 Mg m^−^^3^
Orthorhombic, *P**b**c**a*	Cu *K*α radiation, λ = 1.54187 Å
Hall symbol: -P 2ac 2ab	Cell parameters from 17442 reflections
*a* = 11.900 (2) Å	θ = 3.0–68.2°
*b* = 9.1978 (15) Å	µ = 0.78 mm^−^^1^
*c* = 14.521 (3) Å	*T* = 123 K
*V* = 1589.4 (5) Å^3^	Prism, brown
*Z* = 8	0.50 × 0.40 × 0.40 mm

### Data collection

**Table:**

Rigaku R-AXIS RAPID diffractometer	1359 reflections with *F*^2^ > 2σ(*F*^2^)
Detector resolution: 5.00 pixels mm^-1^	*R*_int_ = 0.018
ω scans	θ_max_ = 68.2°
Absorption correction: multi-scan (*ABSCOR*; Higashi, 1995)	*h* = −14→14
*T*_min_ = 0.566, *T*_max_ = 0.731	*k* = −10→10
18038 measured reflections	*l* = −17→17
1456 independent reflections	

### Refinement

**Table:**

Refinement on *F*^2^	0 restraints
*R*[*F*^2^ > 2σ(*F*^2^)] = 0.044	All H-atom parameters refined
*wR*(*F*^2^) = 0.119	*w* = 1/[σ^2^(*F*_o_^2^) + (0.0661*P*)^2^ + 0.5878*P*] where *P* = (*F*_o_^2^ + 2*F*_c_^2^)/3
*S* = 1.08	(Δ/σ)_max_ < 0.001
1456 reflections	Δρ_max_ = 0.29 e Å^−^^3^
119 parameters	Δρ_min_ = −0.13 e Å^−^^3^

### Special details

**Table:**

Geometry. All e.s.d.'s (except the e.s.d. in the dihedral angle between two l.s. planes) are estimated using the full covariance mat- rix. The cell e.s.d.'s are taken into account individually in the estimation of e.s.d.'s in distances, angles and torsion angles; correlations between e.s.d.'s in cell parameters are only used when they are defined by crystal symmetry. An approximate (isotropic) treatment of cell e.s.d.'s is used for estimating e.s.d.'s involving l.s. planes.
Refinement. Refinement was performed using all reflections. The weighted *R*-factor (*wR*) and goodness of fit (*S*) are based on *F*^2^. *R*-factor (gt) are based on *F*. The threshold expression of *F*^2^ > 2.0 σ(*F*^2^) is used only for calculating *R*-factor (gt).

### Fractional atomic coordinates and isotropic or equivalent isotropic displacement parameters (Å^2^)

**Table:**

	*x*	*y*	*z*	*U*_iso_*/*U*_eq_	
O(1)	0.41204 (11)	1.07612 (13)	0.22608 (7)	0.0476 (3)	
N(1)	0.55271 (11)	0.89513 (13)	0.31064 (8)	0.0327 (3)	
N(2)	0.63438 (13)	0.87983 (16)	0.27341 (9)	0.0441 (3)	
C(1)	0.45523 (13)	0.92398 (16)	0.35238 (10)	0.0336 (3)	
C(2)	0.38659 (14)	1.02931 (17)	0.30396 (11)	0.0390 (4)	
C(3)	0.28687 (14)	1.07339 (17)	0.35513 (11)	0.0406 (4)	
C(4)	0.26340 (13)	1.01769 (18)	0.43965 (12)	0.0419 (4)	
C(5)	0.33263 (12)	0.91102 (16)	0.48560 (10)	0.0344 (3)	
C(6)	0.30613 (13)	0.85591 (18)	0.57389 (11)	0.0389 (4)	
C(7)	0.37387 (14)	0.75293 (19)	0.61482 (11)	0.0395 (4)	
C(8)	0.46903 (13)	0.70173 (19)	0.56895 (11)	0.0388 (4)	
C(9)	0.49762 (13)	0.75479 (17)	0.48334 (10)	0.0343 (3)	
C(10)	0.43117 (12)	0.85986 (16)	0.44128 (10)	0.0310 (3)	
H(1)	0.2371	1.1427	0.3288	0.049*	
H(2)	0.1977	1.0508	0.4703	0.050*	
H(3)	0.2412	0.8901	0.6052	0.047*	
H(4)	0.3558	0.7168	0.6743	0.047*	
H(5)	0.5146	0.6295	0.5971	0.047*	
H(6)	0.5629	0.7195	0.4530	0.041*	

### Atomic displacement parameters (Å^2^)

**Table:**

	*U*^11^	*U*^22^	*U*^33^	*U*^12^	*U*^13^	*U*^23^
O(1)	0.0703 (8)	0.0379 (6)	0.0347 (6)	0.0036 (5)	−0.0079 (5)	0.0033 (4)
N(1)	0.0406 (7)	0.0295 (6)	0.0279 (6)	−0.0016 (5)	0.0014 (5)	0.0001 (4)
N(2)	0.0509 (8)	0.0411 (8)	0.0403 (7)	−0.0027 (6)	0.0117 (6)	0.0025 (6)
C(1)	0.0362 (8)	0.0325 (7)	0.0320 (7)	−0.0001 (6)	−0.0012 (6)	−0.0042 (5)
C(2)	0.0501 (9)	0.0313 (8)	0.0355 (8)	−0.0008 (6)	−0.0102 (7)	−0.0042 (6)
C(3)	0.0414 (8)	0.0361 (8)	0.0442 (9)	0.0067 (6)	−0.0124 (7)	−0.0073 (6)
C(4)	0.0327 (7)	0.0423 (8)	0.0506 (9)	0.0021 (6)	−0.0036 (6)	−0.0167 (7)
C(5)	0.0317 (7)	0.0345 (8)	0.0371 (8)	−0.0045 (6)	−0.0059 (6)	−0.0105 (6)
C(6)	0.0305 (7)	0.0483 (9)	0.0379 (8)	−0.0096 (6)	0.0041 (6)	−0.0150 (7)
C(7)	0.0428 (8)	0.0484 (9)	0.0274 (7)	−0.0132 (7)	0.0011 (6)	−0.0029 (6)
C(8)	0.0388 (8)	0.0428 (9)	0.0349 (8)	−0.0056 (6)	−0.0043 (6)	0.0004 (6)
C(9)	0.0300 (6)	0.0384 (8)	0.0344 (8)	−0.0019 (6)	−0.0001 (6)	−0.0027 (6)
C(10)	0.0308 (7)	0.0336 (7)	0.0288 (7)	−0.0074 (5)	−0.0015 (5)	−0.0071 (5)

### Geometric parameters (Å, °)

**Table:**

O(1)—C(2)	1.2474 (19)	C(6)—C(7)	1.378 (2)
N(1)—N(2)	1.1210 (19)	C(7)—C(8)	1.396 (2)
N(1)—C(1)	1.3355 (19)	C(8)—C(9)	1.378 (2)
C(1)—C(2)	1.449 (2)	C(9)—C(10)	1.390 (2)
C(1)—C(10)	1.448 (2)	C(3)—H(1)	0.950
C(2)—C(3)	1.458 (2)	C(4)—H(2)	0.950
C(3)—C(4)	1.359 (2)	C(6)—H(3)	0.950
C(4)—C(5)	1.444 (2)	C(7)—H(4)	0.950
C(5)—C(6)	1.414 (2)	C(8)—H(5)	0.950
C(5)—C(10)	1.418 (2)	C(9)—H(6)	0.950
			
N(2)—N(1)—C(1)	175.50 (14)	C(1)—C(10)—C(5)	115.66 (12)
N(1)—C(1)—C(2)	113.72 (13)	C(1)—C(10)—C(9)	124.23 (13)
N(1)—C(1)—C(10)	119.70 (13)	C(5)—C(10)—C(9)	120.11 (13)
C(2)—C(1)—C(10)	126.40 (13)	C(2)—C(3)—H(1)	119.2
O(1)—C(2)—C(1)	122.26 (14)	C(4)—C(3)—H(1)	119.2
O(1)—C(2)—C(3)	124.32 (14)	C(3)—C(4)—H(2)	118.1
C(1)—C(2)—C(3)	113.42 (13)	C(5)—C(4)—H(2)	118.1
C(2)—C(3)—C(4)	121.52 (14)	C(5)—C(6)—H(3)	119.8
C(3)—C(4)—C(5)	123.78 (14)	C(7)—C(6)—H(3)	119.8
C(4)—C(5)—C(6)	122.35 (13)	C(6)—C(7)—H(4)	120.0
C(4)—C(5)—C(10)	119.17 (13)	C(8)—C(7)—H(4)	120.0
C(6)—C(5)—C(10)	118.48 (13)	C(7)—C(8)—H(5)	119.6
C(5)—C(6)—C(7)	120.45 (14)	C(9)—C(8)—H(5)	119.6
C(6)—C(7)—C(8)	120.04 (14)	C(8)—C(9)—H(6)	119.9
C(7)—C(8)—C(9)	120.75 (15)	C(10)—C(9)—H(6)	119.9
C(8)—C(9)—C(10)	120.14 (14)		
			
N(2)—N(1)—C(1)—C(2)	29.7 (19)	C(3)—C(4)—C(5)—C(6)	179.60 (15)
N(2)—N(1)—C(1)—C(10)	−145.8 (18)	C(3)—C(4)—C(5)—C(10)	−0.0 (2)
N(1)—C(1)—C(2)—O(1)	7.0 (2)	C(4)—C(5)—C(6)—C(7)	179.26 (15)
N(1)—C(1)—C(2)—C(3)	−173.04 (13)	C(4)—C(5)—C(10)—C(1)	1.5 (2)
N(1)—C(1)—C(10)—C(5)	172.20 (13)	C(4)—C(5)—C(10)—C(9)	−178.53 (14)
N(1)—C(1)—C(10)—C(9)	−7.8 (2)	C(6)—C(5)—C(10)—C(1)	−178.15 (13)
C(2)—C(1)—C(10)—C(5)	−2.6 (2)	C(6)—C(5)—C(10)—C(9)	1.8 (2)
C(2)—C(1)—C(10)—C(9)	177.38 (15)	C(10)—C(5)—C(6)—C(7)	−1.1 (2)
C(10)—C(1)—C(2)—O(1)	−177.92 (14)	C(5)—C(6)—C(7)—C(8)	−0.3 (2)
C(10)—C(1)—C(2)—C(3)	2.1 (2)	C(6)—C(7)—C(8)—C(9)	1.1 (2)
O(1)—C(2)—C(3)—C(4)	179.61 (15)	C(7)—C(8)—C(9)—C(10)	−0.4 (2)
C(1)—C(2)—C(3)—C(4)	−0.4 (2)	C(8)—C(9)—C(10)—C(1)	178.89 (14)
C(2)—C(3)—C(4)—C(5)	−0.6 (2)	C(8)—C(9)—C(10)—C(5)	−1.1 (2)

### Hydrogen-bond geometry (°)

**Table:**

*D*—H···*A*
—···
